# Composite Remineralization of Bone-Collagen Matrices by Low-Temperature Ceramics and Serum Albumin: A New Approach to the Creation of Highly Effective Osteoplastic Materials

**DOI:** 10.3390/jfb15020027

**Published:** 2024-01-23

**Authors:** Vladislav V. Minaychev, Anastasia Yu. Teterina, Polina V. Smirnova, Ksenia A. Menshikh, Anatoliy S. Senotov, Margarita I. Kobyakova, Igor V. Smirnov, Kira V. Pyatina, Kirill S. Krasnov, Roman S. Fadeev, Vladimir S. Komlev, Irina S. Fadeeva

**Affiliations:** 1 Institute of Theoretical and Experimental Biophysics, Russian Academy of Sciences, 142290 Pushchino, Russia; vminaychev@gmail.com (V.V.M.); a.s.senotov@gmail.com (A.S.S.); fadeevrs@gmail.com (R.S.F.); 2 Baikov Institute of Metallurgy and Materials Science, Russian Academy of Sciences, Leninskiy Prospect 49, 119334 Moscow, Russia; teterina_imet@mail.ru (A.Y.T.); smirnova-imet@mail.ru (P.V.S.);; 3 Center for Translational Research on Autoimmune and Allergic Disease—CAAD, Department of Health Sciences, Università del Piemonte Orientale, 28100 Novara, Italy; 4 Research Institute of Clinical and Experimental Lymphology—Branch of the Institute of Cytology and Genetics Siberian Branch of Russian Academy of Sciences, 630060 Novosibirsk, Russia

**Keywords:** biomimetic scaffolds, bone grafts, bioactive materials, remineralization, bone tissue engineering, demineralized bone matrix, coatings, calcium phosphate-albumin composite, bone marrow bio-mimetic

## Abstract

This study examined the effectiveness of coating demineralized bone matrix (DBM) with amorphous calcium phosphate (DBM + CaP), as well as a composite of DBM, calcium phosphate, and serum albumin (DBM + CaP + BSA). The intact structure of DBM promotes the transformation of amorphous calcium phosphate (CaP) into dicalcium phosphate dihydrate (DCPD) with a characteristic plate shape and particle size of 5–35 µm. The inclusion of BSA in the coating resulted in a better and more uniform distribution of CaP on the surface of DBM trabeculae. MG63 cells showed that both the obtained forms of CaP and its complex with BSA did not exhibit cytotoxicity up to a concentration of 10 mg/mL in vitro. Ectopic (subcutaneous) implantation in rats revealed pronounced biocompatibility, as well as strong osteoconductive, osteoinductive, and osteogenic effects for both DBM + CaP and DBM + CaP + BSA, but more pronounced effects for DBM + CaP + BSA. In addition, for the DBM + CaP + BSA samples, there was a pronounced full physiological intrafibrillar biomineralization and proangiogenic effect with the formation of bone-morrow-like niches, accompanied by pronounced processes of intramedullary hematopoiesis, indicating a powerful osteogenic effect of this composite.

## 1. Introduction

Ideal bone substitutes are presented as highly porous materials with high biocompatibility, osteoinductivity, osteoconductivity, and osteogenic potential [1,2,3,4].

In modern orthopaedic surgeries, bone tissue autografts remain the gold standard, as this material already contains all the necessary components for full biontegration of the implanted tissue (living cells, signaling molecules, regulatory extracellular matrix (ECM), etc.). However, well-known complications and inevitable autograft deficiencies have stimulated the development of modern methods of bone tissue engineering, focusing on the creation of bone tissue biomimetics. Such mimetic constructs are required to contain ECM (or its full analog), which simultaneously provides haptotaxis (chemical cues on the surface), durotaxis (mechanical substrate compliance), and topotaxis (geometric features of the substrate) [5], as well as biological and chemical nature-like agents that provide chemotaxis, guided migration, and subsequent differentiation of the patient’s cells. Despite the abundance of synthetic scaffolds, in the above-mentioned context, the demineralized bone matrix has the most pronounced conformity for the provision of topotaxis, haptotaxis, and durotaxis because of its initial optimal geometry, the presence of all necessary signaling functional peptides on the surface of its collagen trabeculae, and because it is a physiological base for the precipitation of amorphous calcium phosphate, resulting in an optimal and specific hydroxyapatite for natural mechanisms of biomineralization. In addition, biological and chemical agents used for chemotaxis and differentiation of the recipient’s cells should also be natural, but remain affordable and controllable in terms of their activity (which is an issue, for instance, in the case of rhBMP-2 or stem cells) [6,7,8,9]. Thus, the utilized bioactive agents can be represented by calcium phosphates preceding the final form of hydroxyapatite (amorphous calcium phosphate—AmP, dicalcium phosphate dihydrate—DCPD, tricalcium phosphate—TCP, octacalcium phosphate—OCP, etc.), as well as bioactive molecules, proteins, and lipids, which participate in the regeneration processes and normal osteogenesis [7,8,9,10].

Considering the deficiency of allogenic tissues in obtaining natural bone substitutes, it appears logical to use demineralized and antigen-free xenogeneic bone tissue [11,12,13]. The demineralized bone matrix consists almost entirely of type I collagen [14,15]. In addition to that, “hole zones” of bone collagen fibrils are not shielded by glycosaminoglycans, leading to nearly non-energy-consuming adsorption of amorphous calcium phosphates with a formation of hydroxyapatite crystals being the terminal and bioinert structure, mechanically stabilizing the supporting bone tissue and presenting the main depot of calcium and phosphates in the organism [16,17]. The triple helix zone of collagen demonstrated a high degree of evolutionary stability, with variations in the amino acid content in different species of mammals not exceeding several percent [18]. It is worth noting that antigenicity and immunogenicity of collagen are caused in the first place by the release of antigenic determinants, which are normally hidden epitopes that interact with antibodies only after the triple helix is unfolded; that is, immunogenic factors can be only damaged and/or denatured collagen [19,20]. Another important consideration is the use of low-temperature calcium phosphates because of the inability of high-temperature synthesis in the body, which would be atypical for the organism and may mediate foreign body reactions and fibrous encapsulation [21,22]. The low-temperature synthesis of calcium phosphate imitates the physiological inorganic components of native bone tissue. At the same time, the use of hydroxyapatite precursors, but not hydroxyapatite itself, appears to be the most logical, as amorphous calcium phosphates are not only used to form tissue-specific hydroxyapatite but also directly drive the differentiation of migrating recipient’s cells [23,24].

Therefore, composite biomimetic bone tissue-like materials may be an effective osteoplastic material as a balanced complex of low-temperature calcium phosphates (precursors of hydroxyapatite) and additional bioactive natural proteins and lipids precipitated on the highly purified intact and non-immunogenic extracellular bone matrix with a preserved fibrillar structure. Such biomimetics of bone tissue should act as a kind of “primers” for the triggering of all processes necessary for bone tissue regeneration but performed directly by the body itself.

In our previous study, we made the first attempt to create a material based on a high purity extracellular demineralized bone matrix with the greatest amount of ultrastructure preservation and the potential to finely regulate the physical and chemical characteristics of low-temperature DCPD deposition [25]. In this work, the efficiency of the proposed approach was investigated in vitro and in vivo by exploiting the precipitation of demineralized bone matrix amorphous calcium phosphate, including its combination with serum albumin.

## 2. Materials and Methods

### 2.1. Cell Cultures and Reagents

MG-63 human osteoblast-like cells were provided by ATCC (Manassas, VA, USA). MycoFluor™ mycoplasma detection kits were purchased from Thermo Scientific (Waltham, MA, USA). Fetal bovine serum (heat-inactivated) was purchased from Gibco (Gibco, Waltham, MA, USA). Cell culture media EMEM and DMEM, lyophilized bovine serum albumin (BSA), antibiotics ciprofloxacin, gentamicin sulfate, fluorescent dyes Bisbenzimide Hoechst 33342, Propidium Iodide, Calcein AM, trypan blue (C.I. 23850) and other chemicals were purchased from Sigma-Aldrich (Milwaukee, WI, USA). Antimycotic fluconazole was purchased from Pfizer (Paris, France). Cell Viability Reagent Alamar-Blue was purchased from Invitrogen (Carlsbad, CA, USA). Ammonium dihydrogen phosphate and calcium nitrate tetrahydrate was purchased from ITW Reagents (AppliChem GmbH, Darmstadt, Germany). Sedative/muscle relaxant Xylazine was purchased from Interchemie werken De Adelaar B.V. (Venray, The Netherlands). Anesthetic Zoletil was purchased from Virbac (Carros, France).

All other reagents used were of analytical reagent quality. The chemical agents were used exactly as provided. All compounds and solvents used in this study were bought commercially and used without additional purification.

### 2.2. Preparation of Collagen Bone Tissue Matrices

Using the epiphyses of the femoral and tibial bones of healthy mature bulls and the author’s method (patent RU 2,686,309 C1, 25 April 2019), demineralized and acellularized bone matrices (DBM) were produced [25]. Briefly, the spongy tissue of healthy mature bulls was subjected to thorough mechanical cleaning and subsequent multi-stage treatment (with periodic vacuuming up to 10–30 mmHg) with 0.5% lipase solution (pH 8.0), a mixture of chloroform and water (1:1), a solution of 20% ethanol, a solution of 0.6 M hydrochloric acid, and a solution of 5% sodium thiosulfate, followed by thorough washing with saline solution, normalization in a buffered saline solution (pH 7.4), freezing, and lyophilization. The main feature of this solution is the production of demineralized bone tissue with a high degree of integrity of the collagen extracellular matrix (absence of damage to the ultrastructure of trabecular matrix collagen), which ensures a high level of bio-integration of this biologically stable scaffold in the recipient’s body and the absence of signs of resorption up to 26 weeks of ectopic implantation.

The DBMs with axial dimensions of 1 × 1 × 0.5 cm were sterilized by antimycotics (fluconazole 0.04 mg/mL) and antibiotics (ciprofloxacin 0.008 mg/mL and gentamicin sulfate 0.02 mg/mL) in sterile phosphate-buffered saline (PBS) for 48 h at 37 °C with constant shaking. After sterilization in PBS with antibiotics and antimycotics, the DBM blocks were washed three times in sterile PBS with a pH of 7.4. The concentrations of calcium and DNA in the samples were determined by spectrophotometry, as previously described [25].

### 2.3. CPs Coating of Demineralized Bone Matrices

The preparation of calcium phosphate coating was carried out under conditions that mimic physiological conditions as much as possible. The investigated samples were obtained as follows. In sterile conditions, samples (10 × 10 × 5 mm) were obtained by cutting bone blocks (DBM) with similar porosity and size. Separate solutions for remineralization were prepared, each with a volume of 30 mL. The components were added in the following order: distilled water, phosphate ion solution (0.588 M NH_4_H_2_PO_4_), bovine serum albumin (BSA) * (4% solution, * for DBM + CaP + BSA samples), then the calcium ion solution (1 M Ca(NO_3_)_2_ × 4H_2_O). The resulting suspensions were rapidly mixed and used to fill fragments of DBM in 50 mL test tubes. The contents of the test tubes were subjected to vacuuming for 30 min at 20 mbar using a Millivac Maxi membrane pump (Millipore, Burlington, MA, USA). After vacuuming, the test tubes were sealed and placed in a shaking incubator at a temperature of 37 °C and 60 RPM.

### 2.4. FTIR Spectroscopy and X-ray Diffraction Analysis

The phase compositions were investigated using a Shimadzu XRD-6000 diffractometer (Shimadzu, Tokyo, Japan) equipped with an programmed imaging system that facilitated visual processing, data collection, and phase identification using a JCPDS 2003 data bank. Powder-dried materials were subjected to X-ray phase analysis for general phase analysis using CuKα monochromatic radiation.

The normalized Chang technique was used to calculate the mass fractions of the samples. The Jana 2006 program was used to fully analyze the diffraction pattern for this purpose (wRp = 1.89%). The JCPDS 2003 data bank descriptions of the corundum number phases and nonoverlapping main peak intensities were used. Lattice constants were calculated in CelRef program (version V3 (2001), INP, Saint-Martin d’Hères, France). using several peaks: (020), (021), (041), (−221), (151). Calculated results are in good agreement with values obtained in Jana 2006 (version 20/02/2023, FZU, Praha, Czesh Republic).

On an Avatar 330 FT-IR spectrometer (Thermo Nicolet Corp., Madison, WI, USA), the tablets’ infrared (FT-IR) spectra were captured in the 4000–400 cm^−1^ wavelength range; 1.0 mg of powder was mixed with 50.0 mg of spectroscopic-grade potassium bromide before the mixture was pressed into a pellet. The pellets were examined at 20 °C in the transmission mode of the main box.

### 2.5. MicroCT and Microstructure Analysis

Microcomputer tomography (microCT) was carried out on the microtomography “SKYSCAN 1275” (Bruker micro-CT, Kontich, Belgium), with a resolution of 4.5 microns, in order to provide a detailed analysis of the morphological and density characteristics of porosity and thickness of materials in the Comprehensive TEX Archive Network (CT-an) program. The images were taken using a 13.76 m voxel size and 0.73° with NReconTM v.1.6.8.0, SkyScan, 2011 (Bruker, Kontich, Belgium). In the reconstruction, ring artifacts and beam-hardening corrections were made. The Data Viewer TM 1.4.4.0 program (Bruker, Kontich, Belgium) was then used to realign the reconstructed images. The quality of the specimens and their interior architectonics were investigated using this technique.

Scanning electron microscopy (Tescan VEGA III, Brno, Czech Republic) and energy-dispersive spectroscopy (EDS; INCA Energy Oxford Instruments, Abingdon, UK) were used to analyze the microstructure and morphology of the sample surfaces and slices. Before analysis, the samples were coated with gold using a Sputter Coater Q150R (Quorum Technologies, Lewes, UK). At pressures of 7.3 10^−2^ Pa in the column and 1.5 10^−1^ Pa in the chamber, surface pictures of the materials were produced. Calcium-to-phosphorus ratios were calculated using energy-dispersive X-ray spectroscopy and Oxford AZ-tecO 4.3 software (Oxford Instruments NanoAnalysis, High Wycombe, UK).

### 2.6. Cell Viability Assay

The MG-63 human osteoblast-like cells for this study were used. There were no signs of mycoplasma infection in the cell cultures. The cells were grown in EMEM with addition of 10% heat-inactivated fetal bovine serum (FBS) and 40 g/mL gentamicin sulfate at 37 °C and 5% CO_2_ in the air. MG-63 cells were seeded at a density of 5 × 10^3^ cells per 100 µL into a 96-well plates (Corning Inc., Corning, NY, USA) of complete growth medium at various calcium phosphate compound concentrations. Following a 24 h cultivation period, the growth medium was changed to 100 µL of medium containing CaP or CaP + BSA at concentrations of 10, 3, 1, 0.3, and 0.1 mg/mL. Cultivation was then carried out further for 24- and 96 h periods. The growth medium used for cell culture under control conditions did not contain CPs. In accordance with the recommended procedure, CaP and CaP + BSA samples were pre-sterilized with 75% ethanol [26].

Alamar-Blue assay was used to measure cell viability following CaP and CaP + BSA incubation. AlamarBlue was administered to the cells at a concentration of 100 µg/mL after 24 and 96 h of incubation. The fluorescence intensity was measured using an Infinity F 200 plate reader (Tecan, Männedorf, Switzerland) at excitation wavelengths of 560 nm and 595 nm after the cells had been cultured for 4 h at 37 C and 5% CO_2_ concentration in the air. The mean fluorescence intensity (MFI) of the generated resofurin product was used to gauge the viability of the cells. Control cells that were not cultured with CaP or CaP + BSA were assumed to have 100% viability. The formula for calculating cell viability after incubation with CaP and CaP + BSA was: cell viability% = (MFI cells after treatment with CaP and CaP + BSA/MFI control cells) × 100%. Additionally, to assess the effect of CaP and CaP + BSA on cell viability, a trypan blue exclusion test was used [27]. Cells were stained with 0.2% trypan blue solution for 3–5 min after 24 and 96 h of incubation with CaP or CaP + BSA. The number of stained and unstained cells was determined by counting on a haemocytometer. The percentages of viable cells were calculated using the following formula: % viable cells = (number of unstained cells/total number of cells) × 100%.

### 2.7. Fluorescence Microscopy and Morphology Analisys

The MG-63 human osteoblast-like cells were used for the morphological analysis. The cells were grown in EMEM with addition of 10% heat-inactivated fetal bovine serum (FBS) and 40 g/mL gentamicin sulfate at 37 °C and 5% CO_2_ in the air. MG-63 cells were seeded at a density of 5 × 103 cells per 100 µL into a 96-well plates (Corning Inc., Corning, NY, USA) of complete growth medium. Following a 24 h cultivation period, the growth medium was changed to 100 µL of medium containing CaP or CaP + BSA at concentrations of 10, 3 and 1 mg/mL. Cultivation was then carried out further for a 96 h period. The growth medium used for cell culture under control conditions did not contain CPs. Hoechst 33,342 (1 µg/mL, blue nuclei of living and dead cells), Propidium Iodide (PI, 1 µg/mL, orange nuclei of dead cells), and Calcein AM (2 mM, green cytoplasm of living cells) were used to assess the morphological status of the cells in culture after 96 h of CPs incubation at 37 °C in a CO_2_ incubator (Binder GmbH, Tuttlingen, Germany). The cell filler plate and samples were placed in a microscope chamber at 37 °C and 5% CO_2_. The stained cell cultures were examined microscopically using a Nikon Eclipse Ti-E microscope (Nikon, Tokyo, Japan).

### 2.8. Animals and Surgical Manipulations

All animal studies followed the Rules for Studies with Experimental Animals (Decree of the Russian Ministry of Health on 12 August 1997; No. 755) and the Directive 2010/63/EU for the protection of animals used for scientific purposes. The Institute of Theoretical and Experimental Biophysics Commission on Biological Safety and Ethics approved this study in February 2018 (Protocol N15/2018).

Eighteen male Wistar rats (two months, 180–200 g) were used for in vivo experiments. The animals were kept separately in temperature-controlled rooms (22 °C), relative humidity 30–60%, 12 h lighting cycle and fed a normal diet, with unrestricted access to food and water (ad libitum). The Guide for the Care and Use of Laboratory Animals (2011) defines appropriate bounds for microclimate parameters in animal rooms. The animals were kept in groups of 3–4 individuals in Type-4 cages (1820 sq. cm) on a bedding consisting of wood chips (SAFE BK8/15, Rosenberg, Germany). Polycarbonate cages are equipped with steel lattice covers with a feed recess, steel feed dividers, and steel label holders. Additionally, animal cages were supplied with materials to enrich the environment, such as yurt-houses made of red transparent polycarbonate (Techniplast S.p.A, Buguggiate, Italy).

The rats were divided into three groups with six rats in each group, and independent replicates were performed for each group (Table 1).

The osteoinductive, osteogenic, and proinflammatory potentials of the samples were evaluated using an ectopic (subcutaneous) implantation model. This model most accurately captures the desired outcomes for confirming the capabilities of materials because it offers findings initiated by the substance alone rather than under the impact of the natural bone microenvironment [25,28,29,30]. Seven weeks was selected as the time frame that corresponds to the full healing of fractured bone. The assessment of the degradation of the materials in dynamics was not carried out because albumin at the concentration used is catabolized by the body in a short time, DCPD resorption by this time was established by us earlier [25], and pure DBM scaffolds obtained by the author’s method were fully integrated into tissues (without any signs of osteoinduction) and were not exposed to resorption up to 26 weeks of ectopic implantation (patent RU 2,686,309 C1, 25 April 2019). Thus, the purpose of implantation for a specified period of seven weeks was to establish the osteogenic potential of the material under ectopic conditions.

The implantation procedures were performed under general anesthesia (xylazine 13 g/kg and zoletil 7 g/kg), as previously described [25]. A heating plate was used to help the animals recover after surgery. The animals’ health was evaluated on a daily basis following implantation.

Seven weeks after implantation, animals from each group were euthanized using the carbon dioxide protocol. Samples of implanted materials with surrounding recipient tissues were fixed for 48 h in neutral buffered formalin at a tissue-fixator volume ratio of 1:30 immediately following humane killing in order to prevent autolysis.

### 2.9. Histological Analysis

Following the completion of fixation, sample fragments were rinsed in distilled water for 3 min to remove excess phosphates before being placed in medium Optimum Cutting Temperature (O.C.T.) Compound Tissue Tek (Sakura, Tokyo, Japan) for at least 12 h. Cryosectioning was used to create cross sections of DBM samples measuring 9 μm (MEV SLEE Medical GmbH, Nieder-Olm, Germany). The samples were stained using a traditional technique that included Hematoxylin and Eosin Y (H&E), alizarin red S (by the McGee–Russell method for calcium deposits [31]), and collagen/non-collagen structures (by Lillie’s trichrome method).

### 2.10. Histomorphometric Analysis

A Nikon Eclipse Ti-E microscope station (Nikon, Tokyo, Japan) and splicing method was used to capture the high-resolution histotopograms of the stained histological samples, which were then processed using the NIS Elements AR4.13.05 (Build 933, Nikon, Tokyo, Japan) program. All images were taken under the same settings, including the same exposure and lighting intensity.

Histomorphometric analysis of the obtained histological images was performed using ImageJ software (version 1.54h, NIH, Bethesda, MD, USA, https://github.com/imagej/ImageJ, accessed on 16 March 2023). The degree of vascularization and mineralization of DBM, DBM + CaP, DBM + CaP + BSA was determined per field of view using. In each group, at least 4 sections were analyzed (n ≥ 4). At least 12 fields per section were used for analysis (n ≥ 12).

Counting the number of vessels in the field of view was used to determine the degree of vascularization. The erythroblastic islands were also calculated quantitatively for each field of view (1 field of view equals 1 sq. mm).

Preprocessing of images to create masks of mineralization areas of DBM + CaP and DBM + CaP + BSA before and after implantation included changing the color balance of the images, followed by their conversion to 8-bit format and the selection of trabeculae or their mineralization zone by setting the appropriate threshold. The area of the trabecular mineralization zone was calculated as a percentage of the area of all trabeculae of DBM.

### 2.11. Statistical Analysis

The means and standard deviations of the results are shown (SD). Each in vitro experiment was performed at least four times (n = 4). The Mann–Whitney U test was used to examine the statistical significance of the differences. The size of the observed effects was assessed using standardized mean difference (Hedges’s g).

Python 3 (ver. 3.10.10, OpenSource PSFL License) in the development environment; Spyder (ver. 5.4.1, OpenSource MIT License) and libraries Pandas (ver. 1.5.2, OpenSource BSD-3-Clause License), and Numpy (ver. 1.24.3, OpenSource BSD-like License), and Scipy (ver. 1.10.0, OpenSource BSD-3-Clause License), and the experimental design and associated statistics (U-test) were completed. The libraries Seaborn (ver. 0.12.2, OpenSource BSD-3-Clause License) and Matplotlib (ver. 3.7.0, OpenSource BSD-3-Clause License) were used to construct plots in Python 3 (ver. 3.10.10).

## 3. Results

### 3.1. Results of Efficiency Evaluation of Bone Matrix Demineralization

After multistep treatment of xenogenic bone matrix (decellularization and de-mineralization of bone tissue protocol), it was found that residual calcium content in the samples did not exceed 0.83 ± 0.41 µg/mg of tissue dry mass, with a residual donor cell DNA content of 2.38 ± 0.76 ng/mg tissue mass, indicating a high degree of purification of the xenogenic collagen bone matrix.

### 3.2. Results of Physico-Chemical Analysis and Morphology

**Figure 1:**
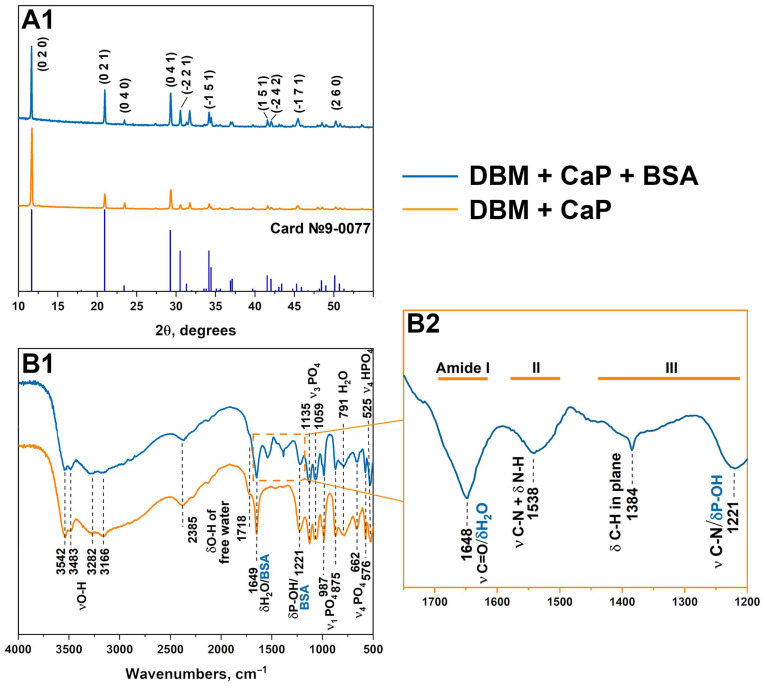
XRD (**A1**) and FTIR (**B1**,**B2**) data of samples DBM + CaP + BSA and DBM + CaP.

The phase compositions of the obtained samples were investigated by X-ray diffraction (XRD) and Fourier-transform infrared spectroscopy (FTIR). Full-profile analysis was performed using Jana 2006 software (version 20/02/2023, FZU, Praha, Czech Republic), and refinement and indexing were carried out using WinXPow software (version 1.2, 27 July 2001, 2000 STOE and Cie GmbH, Darmstadt, Germany). In both experiments, a single-phase crystalline DCPD was obtained, and the diffraction patterns corresponded to card No. 9-0077 of the XRD base ICDD (Powder Diffraction File, Alphabetical Index Inorganic Compounds, Pennsylvania: JCPDS, 1997). The main peaks of DCPD ((020), (021), (041), (−221), etc.) are shown in Figure 1.

The calculated lattice parameters are listed in Table 2. The lattice parameters for the DBM + CaP + BSA samples did not significantly differ from the tabulated values, indicating that BSA was absorbed on the surface of phosphates and no fragments were embedded in the structure [32].

The IR spectroscopy results were in good agreement with the XRD results. The DCPD spectrum has characteristic modes of the HPO_4_^2−^ group at 875 and 987 cm^−1^ (ν1), 987, 1059, and 1135 cm^−1^ (ν3), and 525, 576, and 662 cm^−1^ (ν4). The stretching modes of the O-H bond of lattice water appear at 3550–3160 cm^−1^, bending modes at 1649 cm^−1^. H_2_O libration peak was observed at 791 cm^−1^. The peaks of absorbed water were observed at 2385 and 1718 cm^−1^ [33]. For the DBM + CaP + BSA samples, the modes characteristic of the Ca^2+^-BSA complex appear in the range of 1700–1200 cm^−1^. The peptide bonds of BSA exhibit three bands in the carbonyl absorption region, known as amides I, II, and III (indicated by blue lines in Figure 1) [34]. The band at 1648 belongs to the lattice water and amide I (C=O stretching), amide II appears as a mode at 1538 cm^−1^ (C–N stretching coupled to N–H bending), and peaks of amide III are observed at 1384 cm^−1^ (C–H in-plane bending vibration) and 1221 cm^−1^ (C–N stretching) [35,36]. The peak at 1221 cm^−1^ also corresponds to P-OH bending.

**Figure 2:**
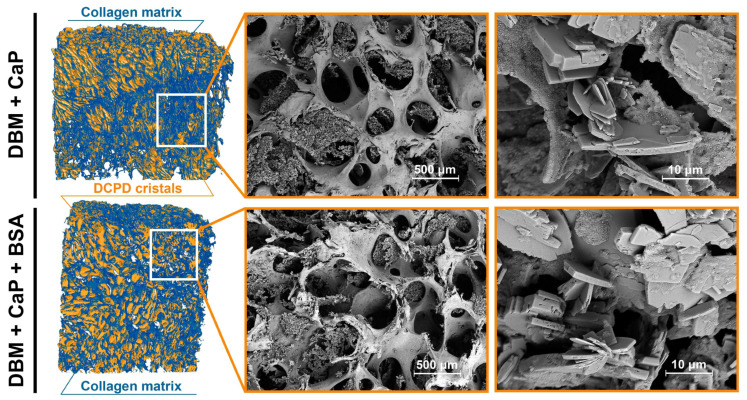
MicroCT—images and SEM of the surface of the samples.

Scanning electron microscopy data confirmed the results obtained by spectral methods. Figure 2 shows that typical brushite particles are deposited in the DBM porous matrix. DCPD particles are represented by a lamellar form that is characteristic of calcium phosphate. The particle size varied from 5 µm to 35 µm. According to the MicroCT data, the inclusion of BSA in the coating composition led to a more uniform distribution on the surface of the sample pores. Violation of the matrix and destruction of the walls during the deposition process were not observed.

### 3.3. In Vitro Cytocompatibility Evaluation Results

**Figure 3:**
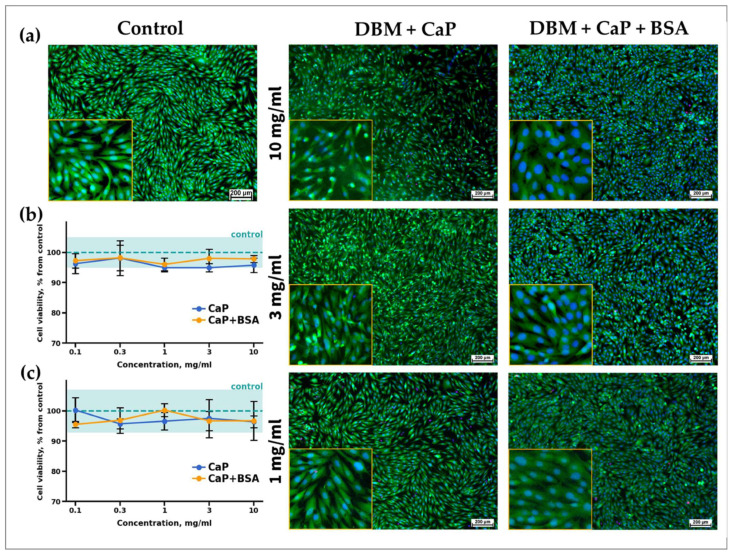
Results of morphological analysis (**a**) and cell viability assays (**b**,**c**) of CaP and CaP + BSA samples. Cell viability of MG-63 cells after 24 h (**b**) and 96 h (**c**). Fluorescence micro-graphs of MG-63 cells after 96 h of cultivation with different concentrations of CaP and CaP + BSA (**c**); live cells are stained with Calcein AM (green), dead cells are stained with PI (red), nuclei of living and dead cells are stained with Hoechst 33,342 (blue); fluorescent microscopy.

The influence of CaP and its complex, CaP + BSA, on MG-63 cell viability was investigated. It was shown that the viability of cells incubated with CaP, as well as with CaP + BSA, did not differ from the control cells incubated without calcium phosphates in all the ranges of studied concentrations and experimental time points (24 and 96 h) (Figure 3a,b). Additionally, the absence of dead cells+ stained with trypan blue was found at all concentrations.

Morphological analysis of MG-63 cells cultivated with CaP and CaP + BSA confirmed these results. Figure 3a shows microscopic images of cells cultivated with CaP and CaP + BSA at maximum concentrations of 10, 3, and 1 mg/mL. The absence of dead cells stained with PI was observed in all concentrations. Moreover, cells cultivated with calcium phosphates had a normal fusiform-elongated morphology, which indicated the absence of calcium phosphate-related stress. Thus, the obtained results indicate the cytocompatibility of the studied calcium phosphates and their complexes with BSA.

### 3.4. Results of the Assessment of Biocompatibility, Osteoconductive, and Osteoinductive Potential of Sammples In Vivo

#### 3.4.1. DBM Implantation Results

**Figure 4:**
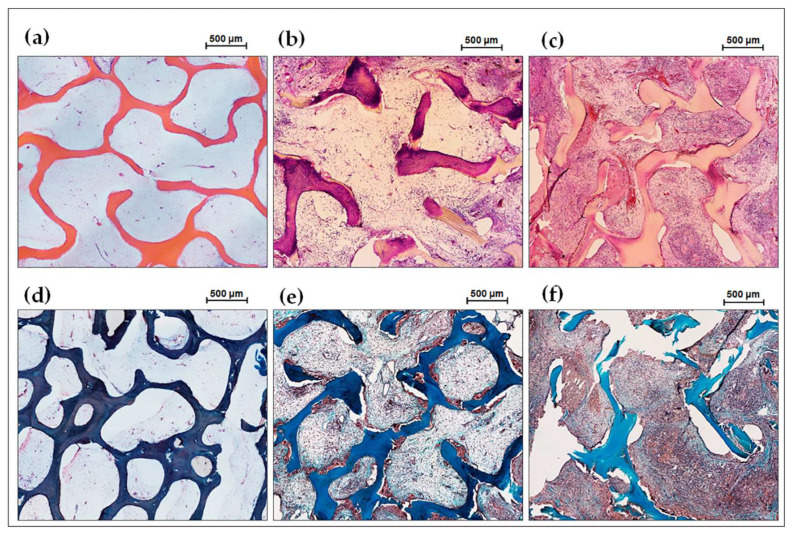
Histological analysis of the implanted DBM specimens of control and experimental groups. (**a**,**d**) DBM without remineralization; (**b**,**e**) DBM + CaP specimens; (**c**,**f**) DBM + CaP + BSA specimens. (**a**–**c**) Haematoxylin & Eosin staining (H&E, cell nuclei are stained blue, erythrocytes are stained red, matrix components are stained pink); (**d**–**f**) Lillie’s trichrome staining (collagen is stained blue, venous sinuses and neocollagenous tissues are stained red, cell nuclei are stained violet-brown); Light microscopy.

**Figure 5:**
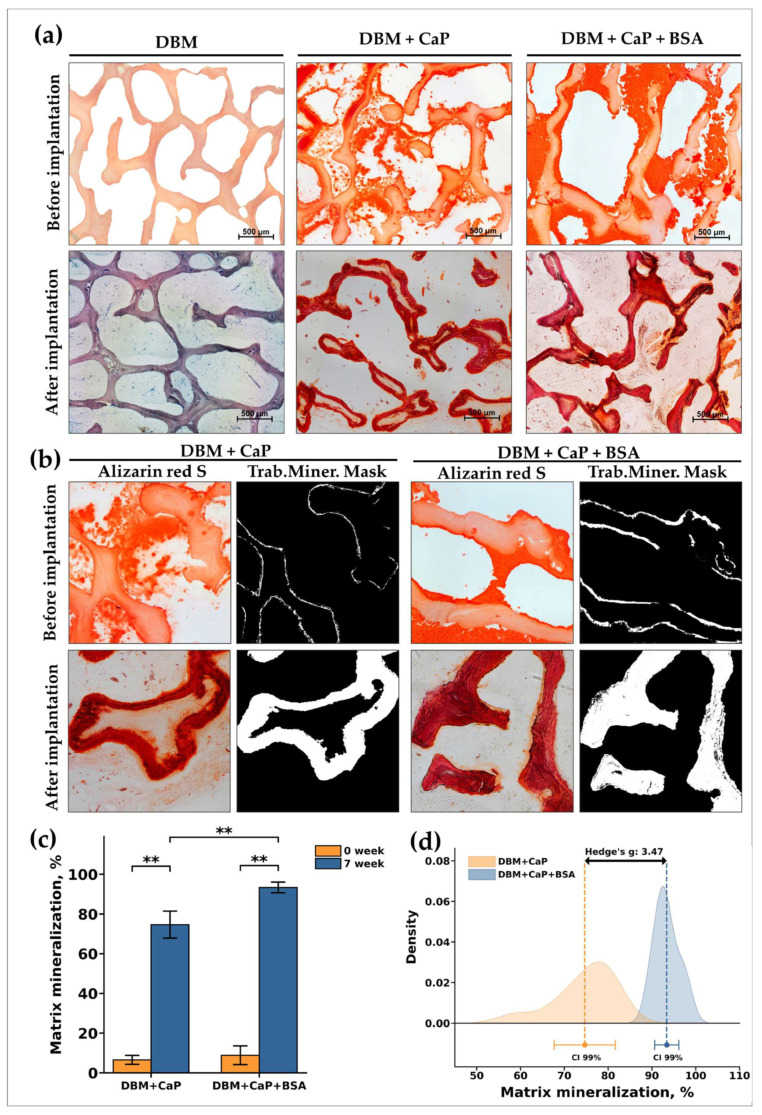
Histological analysis of the implanted DBM samples of control (DBM) and experimental (DBM + CaP and groups. (**a**) DBM—samples of DBM without remineralization (top) and after implanatation (down); DBM + CaP—samples of DBM after remineralization by only CaP and samples of this group after implanatation; DBM + CaP + BSA—samples of DBM after remineralization by CaP + BSA and samples of this group after implanatation; Alizarin red S staining (calcium depositions are stained orange-red), (**a**) due to the absence of the staining by Alizarin red S, the specimens were stained with Toluidine blue for visualization (for DBM after implantation); Light microscopy. (**b**) Results of morphological comparative analysis of the degree of intra-trabecular mineralization of DBM + CaP and DBM + CaP + BSA samples after demineralization and remineralization procedures (top) and after implantation (down): First and third rows—alizarin red S stain, the second and fourth rows—masks of directly intra-trabecular mineralization of DBM obtained using ImageJ software (version 1.54h). (**c**) Results of measuring the area of intra-trabecular mineralization of DBM into DBM + CaP and DBM + CaP + BSA samples. (**d**) Results of comparing the intensity of intra-trabecular mineralization of DBM + CaP and DBM + CaP + BSA samples. Effect size is shown using the standardized mean difference (Hedges’s g). Hedges’s coefficient g ≥ 0.80 indicates a large effect. **—statistically significant differences between the comparison groups, *p* < 0.01. CI—confident interval.

The high biocompatibility of the implanted DBM and the absence of its osteoinductive and osteogenic potential were defined after a 7-week ectopic implantation (Figure 4a,d). Preservation of the collagen matrix after decellularization and demineralization of bone tissue was indicated by the absence of both enzymatic and cell-mediated resorption of the implanted DBM. No leukocyte or foreign-body giant cell (FBGC) invasion was observed in the implanted material, as well as degenerated mast cells in or around the specimens. Signs of neocollagenesis were extremely poor, were observed only at the periphery of the sample, and were initiated by the remodeling processes of the peri-implant tissue (influence of the host tissue), but not by the DBM itself (Figure 4d). Additionally, there was weak growth of reactively altered connective tissue inside the specimen from the periphery of the peri-implant tissue. Complete involution of the fibrous capsule by the studied implantation period was identified, which evidences the bio-integration of the implanted material. However, no signs of DBM mineralization were found, indicating the absence of both synthetic and mineralizing activities of osteoblasts (Figure 4d and Figure 5a (DBM)). Therefore, pure demineralized collagenous bone matrix is a highly biocompatible, non-immunogenic, osteoconductive, and biologically stable scaffold with no osteoinductive and osteogenic properties.

#### 3.4.2. DBM + CaP Implantation Results

In contrast to the DBM specimens, in the implantation of DBM + CaP, a different pattern was observed (Figure 4b,e). In this group, a high degree of intra-trabecular biomineralization without any signs of mineralized matrix degradation by osteoclasts was observed, that is, the nature of biomineralization was purely physiological (Figure 5a,b (DBM + CaP)). Biovisualization of the obtained histological images showed that the grade of intra-trabecular mineralization of the DBM was >70% (Figure 5c). In addition, there were no signs of synthetic activity of cells migrating in the matrix, and the cell response was steady and balanced (Figure 4e). At the same time, no negative adipocyte differentiation or positive osteoblast differentiation was observed (synthetic phenotype) (Figure 4b,e). It seems that in this pattern of remineralization, the matrix is accepted by the body as its own, and mineralization is carried out passively, with no mineralizing osteoblasts taking part. In addition, among the migrated cells, there were no histio-lymphocyte cells, mast cells, or FBGC (Figure 4b). Involution of the fibrous capsule by the observation period was also completed, which indicated the pronounced biocompatibility of the studied materials. Thus, remineralized by the proposed approach, DBM is an efficient material with a high degree of osteointegration and is a full-fledged osteogenic primer to initiate further regenerative processes in peri-implant tissue.

#### 3.4.3. DBM + CaP + BSA Implantation Results

A remarkable pattern was found after seven weeks after implantation of the DBM + CaP + BSA specimens. The materials of this group were completely and physiologically intra-trabecularly mineralized, and biovisualization of the obtained histological images showed that the degree of intramolecular mineralization of the bulk DBM collagen was almost 90% (Figure 5a,b (DBM + CaP + BSA); Figure 5c).

These results (especially the effect size Hedges’s g, 3.47) indicate the pronounced ability of the material to promote full-fledged intra-trabecular mineralization (Figure 5d).

**Figure 6:**
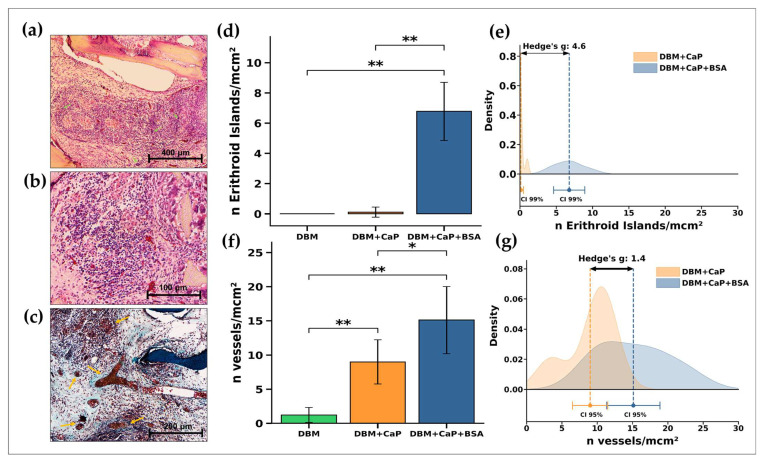
Histological and quantitative analysis of the implanted samples in the DBM + CaP + BSA group. (**a**,**b**) Hematoxylin & Eosin staining (H&E, cell nuclei are stained blue, erythrocytes are stained bright red, stroma components are pink, green arrows indicate erythroblastic islands); (**c**) Lillie’s trichrome (collagen is stained blue, aggregations of synthesizing cells are stained red, cell nuclei are violet-brown, yellow arrows indicate mature definitive vessels); Light microscopy; (**d**) Results of measuring the area of intra-trabecular mineralization of DBM into DBM + CaP and DBM + CaP + BSA samples. (**d**) Results of a quantitative comparative analysis of the number of formed erythroid islands and (**f**) the number of newly formed vessels in DBM + CaP and DBM + CaP + BCA samples. (**e**) Results of comparing the intensity of formation of erythroid islands and (**g**) newly formed vessels in DBM + CaP and DBM + CaP + BCA samples. Effect size is shown using the standardized mean difference (Hedges’s g). Hedges’s coefficient g ≥ 0.80 indicates a large effect (**b**). *—statistically significant differences between the comparison groups, *p* < 0.05. **—statistically significant differences between the comparison groups, *p* < 0.01. CI—confident interval.

The specimens of this group demonstrated extraordinarily active processes of chemo- and haptotaxis, migration, intense proliferation, and synthetic activity of cells inside the implanted materials (Figure 4c,f and Figure 6). As a result, it was observed that the material’s structure included pronounced extramedullary erythropoiesis-like processes (the samples were “sprinkled” of erythrocytes) along with the formation of multicellular endosteal bone marrow-like niches with a high concentration of myeloid precursors, erythroblastic island-like structures, venous sinuses, and mature definitive vessels [37].

At the same time, there were no signs of proteolytic and cell-associated matrix degradation, or its destruction by aseptic utilization calcinosis, that is, this material, despite the active cell response, was indeed favored by the body. In contrast to our previous results, where we observed the indications of pronounced synthetic activity of osteoblasts and DBM remodeling in conformity with the host body, in this case, the matrix itself did not undergo remodeling and was accepted in full, and the synthetic cell activity was mostly connected to the formation of bone marrow-like structures and a complete vascular network. At the same time, given that the newly formed multicellular intertrabecular structures contained relatively large newly formed definitive blood vessels, it can be assumed that immature osteoblasts and M2 macrophages differentiated on the matrix actively produce angiogenic factors [38,39,40,41]. In addition, no negative signs of alterations in healthy tissues surrounding the implant or ectopic aseptic calcification of the peri-implant tissue were found, which indicates a strict material-associated localization of the identified effects.

Through high magnification, Figure 6 shows that, as in a typical scenario, the formations resembling multicellular endosteal bone marrow niches have been identified in the center of the intertrabecular space (Figure 6a,b; green arrows indicate erythroblastic islands) and are surrounded by relatively “calm” non-inflammatory cells. Non-bone collagenous matrix formation is primarily observed for the newly formed sinuses and definitive blood vessels (Figure 6c; yellow arrows). Quantitative measurements of the number of erythroid islands inside the implanted samples revealed a significant difference (*p* < 0.01) between all the groups studied (Figure 6d,f). When comparing the number of newly developed vessels, a significant difference (*p* < 0.01) was also observed in relation to the control (DBM) and between the DBM + CaP and DBM + CaP + BSA groups (*p* < 0.05).

These findings (especially the effect size Hedges’s g, 4.6) represent the strong osteogenic effect of the DBM + CaP + BSA samples in ectopic model implantation. In addition, a relatively high effect size index (Hedges’s g, 1.4) for the number of newly formed vessels also indicates a pronounced proangiogenic effect of the DBM + CaP + BSA samples, which is a very important indicator for the successful bio-integration of bone substitutes.

Thus, DBM remineralized by low-temperature CaP and serum albumin exhibits a high degree of bio-integration and is an extremely bioactive osteogenic material. Based on the results obtained, we can assume that these materials may be fully integrated into the recipient’s bone tissue and initiate further regenerative processes, localizing “on itself”.

## 4. Discussion

The data obtained indicate that the biomimetic approach in the creation of osteoplastic materials is promising and should be based on three main “pillars”: (1) the use of a highly purified non-immunogenic extracellular bone matrix with preserved fibrillar structure and spatial architectonics, (2) the use of low-temperature calcium phosphate compounds as precursors of hydroxyapatite, and (3) the use of natural bioactive agents necessary for bone tissue regeneration.

The exploitation of an intact demineralized bone collagen matrix is of utmost importance because this material can be completely purified and controlled for infections. At the same time, the intact extracellular bone matrix is non-immunogenic and can therefore undergo full remodeling and be incorporated into the body without the stage of intermediate resorption, which can significantly reduce the time of bone tissue regeneration.

Although various bone structures differentiate depending on the type of bone tissue (compact, spongy, etc.), the structure of the mineralized fibrils is conservative; thus, it acts as a universal elementary building block of bone [42]. Therefore, the proposed approach can be extended to other types of demineralized bone tissues to create a wider range of osteogenic materials.

At the same time, such an active cellular response on DBM + CP + BSA materials requires a separate and thorough study to determine whether this approach is acceptable for clinical use. For instance, these materials are hardly applicable in traumatology and maxillofacial surgery, where the formation of bone tissue with potential bone marrow is undesirable. It may be tentatively assumed that the use of such bioactive materials in spinal surgery or surgical correction of non-unions may be useful because such materials can simultaneously be both an effective osteogenic scaffold and a source of progenitor cells without additional costs for preliminary isolation, replication, and introduction of isogenic progenitor cells of the recipient in the scaffold.

On the other hand, as described by Torisawa et al. (2014), obtaining bone marrow mimetics for studying hematopoietic niche physiology in vitro [43] points to the fact that the approach with added serum albumin used in our work may both decrease costs and increase the production efficiency of “bone marrow-on-a-chip” constructs in vitro, which also requires additional detailed investigations.

Interesting results are presented by data on the implantation of a material remineralized with albumin, which makes us appreciate this simple protein as a promising tissue engineering material.

Albumin is the dominant plasma protein, constituting approximately 50% of the total protein concentration in blood plasma and up to 75% of colloidal activity. Albumin is a monomeric multidomain macromolecule that determines the oncotic pressure of plasma and the distribution of fluid between organs and tissues of the body, according to the classical Starling principle [44,45,46]. Under physiological conditions, there is a net movement of albumin from the intravascular to the interstitial space and back through lymphatic vessels [47]. Simultaneously, albumin itself can directly affect the integrity of blood vessels by binding to the interstitial matrix and subendothelium and changing the permeability of these layers for large molecules and solutes [48,49].

The concentration of albumin in blood plasma is approximately 0.6 mM (4% *wt*/*v*), but for clinical use, even 20% (*wt*/*v*) solutions are utilized. Albumin is a classic pleiotropic protein that performs several important functions in the body. Under physiological conditions, human serum albumin (HSA) has the exceptional ability to bind ligands, providing a depot and carriage of endogenous and exogenous compounds such as metal ions, cholesterol [50], thyroxine [51], bilirubin and bile acids [52,53,54], nitric oxide [55], amino acids, and fatty acids [56]. In addition, almost 35 proteins and many peptides are associated with HSA (e.g., angiotensinogen, apolipoproteins, ceruloplasmin, clusterin, haemoglobin, plasminogen, prothrombin, and transferrin) [57], while the fraction of such peptides and proteins are collectively called “albumin” [58]. In addition, after acute hemolysis (due to trauma or post-ischemic reperfusion), albumin binds to the released heme and promotes its transfer to hemopexin, which provides receptor-mediated reuptake of heme by parenchymal liver cells [57,59] and which may be important for orthopaedic surgery and traumatology.

In addition, albumin can bind to almost all known drugs, as well as many nutraceuticals and toxic substances, largely determining their pharmacokinetics and toxicokinetics [60,61]. Albumin is known to provide the highest antioxidant capacity in human plasma. Additionally, the unprotected cysteine residue at position 31 contains reduced sulfhydryl groups, which are mostly obtained from extracellular sources by albumin. These sulfhydryl groups, often known as thiols, actively scavenge reactive oxygen and nitrogen species (ROS and RNS), particularly superoxide hydroxyl and peroxynitrite radicals [45,62]. By binding to free Cu^2+^, an ion known to be particularly crucial for speeding free radical formation [57], albumin may further reduce the production of these reactive molecules. Albumin also plays a role in regulating immunological responses [45], neutralizing and removing potential toxins [57,61], and exhibiting (pseudo)enzymatic properties and peroxidase activity [63] toward lipid hydroperoxides [44,64,65,66,67,68,69,70,71,72,73].

The role of albumin in calcium distribution is important. Normally, it is albumin that is an important carrier of Ca^2+^ in blood plasma, however, the affinity of albumin for Ca^2+^ binding is relatively weak (Kd 0.67 mM, Ka = 1.5 × 10^3^ M^−1^), and only about 45% of 2.4 mM circulating Ca^2+^ binds to albumin [74]. It has long been believed that albumin transports Ca^2+^ through carboxylate side chains on its surface [75,76]. In 1971, Pedersen showed that calcium can bind reversibly to serum albumin via 12 ± 1 independent binding sites with an association constant of 90–100 L/mol at 37 °C in unbuffered solutions at pH 7.4, and an ionic strength of 0.15–0.16 [77]. Despite the work of Pedersen, only three selective Ca^2+^-binding sites in BSA were identified in 2012, all of which are located in domain I [78]. Only recently [79], almost 50 years after Pedersen’s work, 19 free and 11 stable bound Ca^2+^ docking sites (including the original three from the crystal structure) were additionally revealed, and a calcium-dependent change in albumin conformation was identified. This work clearly shows that the amount of Ca^2+^ ions binding to BSA increases with an increase in calcium concentration with a logarithmic-type dependence. However, in the proposed approach, the material worked not only as a calcium-binding and antioxidant protein, but also as a powerful osteogenic calcium–protein complex.

Based on the results of a literature analysis, it was revealed that albumin is physiologically present in the bone tissue and is the first protein secreted by bone cells in the case of bone damage. To obtain a graft that is very similar to the native tissue, it is necessary to replenish the albumin content in bone grafts [80]. It was also shown that during the healing of fractures in the femoral-diaphyseal tissues of rats, the expression of albumin significantly increased in the area of the fracture, while the content of albumin during cultivation significantly increased in the presence of the parathyroid hormone (1–34), IGF-1, and zinc acexamate [81]. Albumin has also been shown to be expressed in osteoblasts and plays a role in regulating the expression of Runx2 or alpha1(I) collagen mRNA, which may be mediated by the intracellular mitogen-activated protein kinase (MAP-kinase) cascade in osteoblasts [82]. The number of osteoblast cells significantly increased when cultured in the presence of albumin (1.0 mg/mL) in vitro for 24–72 h, whereas this effect of albumin was completely eliminated in the presence of PD98049, staurosporine, or dibucaine, which are inhibitors of various protein kinases, and cycloheximide or 5,6-dichloro-1-beta-D-ribofuranosylbenzimidazole (DRB), which are inhibitors of transcriptional activity [83].

Many studies have also shown that the application of an albumin coating on the surface of hyaline cartilage xenografts causes a weakening of immune and inflammatory responses at the cellular, protein, and gene levels, and significantly fewer inflammatory cells (including neutrophils, macrophages, and lymphocytes) in coated xenogeneic materials, accompanied by significantly lower expression of genes encoding inflammation-associated cytokines, including MCP-1, IL-6, and IL-1β [84]. It has also been shown that albumin coatings improve bio- and immune compatibility, tissue formation, corrosion resistance, and antibacterial and anti-clotting properties of materials [85,86,87,88,89,90,91,92]. Albumin has been described in most studies as a protein that inhibits cell adhesion on inert surfaces; however, it is a potent cell adhesive in more physiological scaffolds such as mineralized bone allografts [93,94,95].

Kang et al. [96] showed that Ad-MSCs from adipose tissue showed a more homogeneous distribution and osteogenic differentiation on a porous albumin scaffold with collagen I gel than without collagen I gel. The mineralization of the extracellular matrix and ALP activity in the construct with type I collagen were significantly higher than in the construct without type I collagen (*p* < 0.05). It has been demonstrated that the use of collagen I gel and serum albumin scaffolded together improves osteogenic development and uniform dispersion of Ad-MSCs. Another study [89] found that albumin-coated materials attracted approximately two-fold as many cells as uncoated materials after incubation with MSCs in vitro.

Several studies have shown that albumin improves biointegration by improving the adhesion and proliferation of MSCs on mineralized human bone allografts and stimulating regeneration processes in peri-implant tissue [97,98,99,100,101]. In a double-blind randomized trial [102] it was demonstrated that albumin-coated grafts had the lowest level of postoperative pain, and after 6 and 12 weeks, there were signs of tissue remodeling, while uncoated xenografts were still separated from the host bone; after a year of implantation, complete remodeling and integration with the natural trabecular structure were revealed. Another study [97] focused on the co-cultivation of hADSCs and monocytes in vitro and showed that monocytes have the ability to degrade uncoated bovine bone pellets and that the albumin coating protects such grafts from degradation. Simultaneously, for samples coated with albumin, a significant decrease in the production of ROS and RNS and mitigation of gene expression of mitochondrial electron transport chain complexes was observed. Among the five complexes of the electron transport chain, the sites with the highest ability to produce ROS are complexes I and III [103]. Complex I produces ROS in the matrix, whereas complex III produces ROS both in the matrix and on the inner side of the membrane [103,104]. Moderate levels of mitochondrial ROS have recently been found to directly stimulate the production of proinflammatory cytokines, thus regulating the inflammatory response [105]. Cytokine analysis showed that the cultivation of stem cells and monocytes on albumin-coated grafts led to an increase in the levels of targeted cytokines (HGF, PGE-2, and IL-10) compared with uncoated xenografts, even under conditions that simulate inflammation [97]. Interestingly, the albumin coating was effective only on mineralized allogeneic human bone materials and demineralized bovine bones, but not on hydroxyapatite scaffolds [106,107]. Another study [108] showed that albumin adsorbed on hydroxyapatite promotes significantly higher levels of cell adhesion than albumin adsorbed on control surfaces.

Several studies have shown the antimicrobial properties of albumin coating by preventing bacterial adhesion on the surface of implants coated with albumin [109,110,111]. Albumin also has antithrombotic and anticoagulant effects, possibly owing to its ability to bind to nitric oxide (NO) to form S-nitrosothiols [112]. This inhibits the rapid inactivation of NO and prolongs its anti-aggregation effect on platelets [55,113]. Preliminary application of albumin to the implant surface (“albumin passivation”) effectively prevents platelet activation by creating a thin protein layer on the surface, which increases the hydrophilicity of the surface and prevents a biological reaction after contact with the blood of a hydrophobic material [92,114,115]. It has been demonstrated that materials with adsorbed natural albumin reduce the quantity of adhering platelets and their activation on surfaces. However, platelets were able to fully adhere and activate the modified albumin layer when the albumin structure was changed by crosslinking [88].

It has also been shown that the activity of the oxidative burst of neutrophils is noticeably lower when incubated with albumin, but much higher when incubated with artificial colloids and crystalloids [116]. The pulmonary release of neutrophils in response to exposure to cytokines associated with the pathophysiology of critical diseases, particularly TNF, and elements of the complement system, such as C5A, have also been demonstrated to be suppressed by HSA. Human serum albumin also suppressed TNF-induced neutrophil dissemination and the corresponding drop in cAMP in a selective and reversible manner [117].

All the above studies show that albumin, along with all the previously mentioned properties, can also have pronounced positive effects, including (immunomodulatory functions) which are crucial for healing and tissue regeneration [63]. Thus, based on literature analysis and our data, we can assume that our proposed method of remineralizing highly purified DBM using low-temperature HAp precursors and serum albumin may be an effective and promising approach for obtaining osteoplastic materials with a pronounced osteogenic effect.

In summary, we can assume that our proposed approach is promising, but to confirm this, it is necessary to conduct an additional cycle of studies, including assessing the effect on the biomineralization process of the DPCD or other HAp precursors coating methods, identifying the optimal ratios of organic and inorganic compounds for bone restoration, identifying the ability of composites to maintain the viability of isogenic recipient cells or growth factors, and identifying deadlines for the resorption of materials, as well as the patterns of fibrogenesis and angiogenesis. The establishment of regenerative potential will be performed primarily using orthotopic implantation of materials into critically dimensional defects of the rat skull, as seen in the work by Paulo et al. (2011) [118], or the rabbit scapula, as seen in the work by Hruschka et al. (2017) [119], which will be performed in our future studies.

## 5. Conclusions

Composite bone tissue-like biomimetic constructs may be highly effective osteoplastic materials. These materials are required to contain ECM (or its full analog), which simultaneously provides haptotaxis, durotaxis, and topotaxis as well as biological and chemical nature-like agents that provide chemotaxis, guided migration, and subsequent differentiation of the patient’s cells. In addition, these biomimetic constructs must include low-temperature calcium phosphates (precursors of hydroxyapatite), bioactive natural proteins, and lipids that precipitate on the extracellular bone matrix. Such composite biomimetics should act as a kind of “primers” for triggering all processes necessary for bone tissue regeneration but should be performed directly by the organism itself. In the present study, the efficiency of a similar approach was investigated. The results showed pronounced biocompatibility and strong proangiogenic and osteogenic effects for both DBM + CaP and DBM + CaP + BSA, but more pronounced effects for DBM + CaP + BSA with the formation of bone-morrow-like niches in this composite. Thus, it can be stated that the use of a triad in the form of an intact demineralized bone matrix and low-temperature calcium phosphate compounds (HAp precursors) in combination with albumin is a promising and inspiring approach that can provide osteoinductive, osteoconductive, and angiogenic potentials, biological safety, easy market availability, long shelf life, and reasonable costs.

## Figures and Tables

**Table 1:** Groups and scheme of experiment.

Group #	Samples/Group	Animal Numbers
1	DBM	6 (1–6)
2	DBM + CaP	6 (7–12)
3	DBM + CaP + BSA	6 (13–18)

**Table 2:** Calculated lattice constants of samples.

	a, Å	b, Å	c, Å	α, °	β, °	γ, °	V, Å^3^
Card №9-0077	6.36	15.19	5.82	90.00	118.50	90.00	493.93
CaP	6.362 (3)	15.1609 (24)	5.8083 (14)	90.00	118.545 (21)	90.00	492.13 (18)
CaP + BSA	6.3610 (20)	15.168 (3)	5.8121 (12)	90.00	118.571 (24)	90.00	492.47 (18)

## Data Availability

Data available on request from the authors.
